# Treatment and outcomes of patients with light chain amyloidosis who received a second line of therapy post autologous stem cell transplantation

**DOI:** 10.1038/s41408-022-00655-z

**Published:** 2022-04-11

**Authors:** Abdullah S. Al Saleh, Mohammad S. Ebraheem, M. Hasib Sidiqi, Angela Dispenzieri, Eli Muchtar, Francis K. Buadi, Rahma Warsame, Martha Q. Lacy, David Dingli, Wilson I. Gonsalves, Taxiarchis V. Kourelis, William J. Hogan, Suzanne R. Hayman, Prashant Kapoor, Shaji K. Kumar, Morie A. Gertz

**Affiliations:** 1 Division of Hematology, Department of Internal Medicine, Mayo Clinic, Rochester, Minnesota USA; 2 College of Medicine, King Saud bin Abdulaziz University for Health Sciences, Riyadh, Saudi Arabia; 3 King Abdullah International Medical Research Center, Riyadh, Saudi Arabia; 4 Division of Hematology & HSCT, Department of Oncology, King Abdulaziz Medical City, Ministry of National Guard-Health Affairs, Riyadh, Saudi Arabia; 5 Department of Hematology, Fiona Stanley Hospital, Perth, WA Australia

**Keywords:** Haematological diseases, Medical research

## Abstract

We retrospectively reviewed 292 patients who received a second line of therapy post ASCT for their light chain amyloidosis. Most patients (40%) were treated with an alkylator + PI ± dex or PI ± dex followed by an alkylator + 2nd-gen IMiD ± dex or 2nd-gen IMiD ± dex (26%), an alkylator ± steroid or steroid monotherapy (19%), a 2nd-gen IMiD + PI ± dex (6%), an alkylator + thalidomide ± dex (5%), or daratumumab-based therapy (4%). The rate of CR or VGPR was 70% among the daratumumab-based group, 62% in the alkylator + PI ± dex or PI ± dex group, 55% in the alkylator + 2nd-gen IMiD ± dex or 2nd-gen IMiD ± dex group, 47% in the 2nd-gen IMiD + PI ± dex group, 24% in the alkylator ± steroid or steroid monotherapy group, and 18% in the alkylator + thalidomide ± dex group. The median OS was NR for the 2nd-gen IMiD + PI ± dex group and the daratumumab group, 130.4 months in the alkylator + 2nd-gen IMiD ± dex or 2nd-gen IMiD ± dex group, 100 months for the alkylator + PI ± dex or PI ± dex group, 36 months for the alkylator ± steroid or steroid monotherapy group, and 21 months for the alkylator + thalidomide ± dex group (*P* < 0.0001). The median OS was 100 months in patients who received melphalan 200 mg/m^2^ compared to 41 months in the 140 mg/m^2^ group (*P* < 0.0001). In conclusion, patients receiving novel therapy post ASCT and melphalan conditioning dosing at 200 mg/m^2^ at diagnosis had better outcomes.

## Introduction

Immunoglobulin light chain (AL) amyloidosis is a clonal plasma cell disorder, in which misfolded insoluble protein fibrils accumulate in tissues leading to damage and malfunction of the affected organs. The incidence is estimated to be three to five patients per million per year [1]. The treatment of AL amyloidosis is mainly directed against the clonal plasma cells. Autologous stem cell transplant (ASCT) has improved the survival of AL amyloidosis [2]. However, not all patients are eligible for ASCT and other plasma cell-directed therapy has been used including the use of immunomodulatory drugs (IMiDs), proteasome inhibitors (PIs), alkylators, and CD38 monoclonal antibodies, such as daratumumab.

A significant number of patients relapse after their first line of treatment and the management of these patients can be challenging. The choice of therapy depends on multiple factors including organ involvement, comorbidities, performance status, and previous therapy received. There is limited published data on the best approach for managing patients with AL amyloidosis who relapse after their first therapy, especially for those who had ASCT as their first line. Browing et al. described 82 patients who had a relapse of their AL after ASCT [3]. Most patients received a bortezomib-based (41%) or lenalidomide-based treatment (20%) and the median overall survival (OS) for the whole cohort was 8.5 years. However, details regarding the specific regimens and survival based on the different regimens were not reported. In a report from Mayo Clinic [4], 366 patients with relapsed AL were identified and their treatment and outcomes were described. However, only 108 patients received ASCT as their first line of therapy. Warsame et al. also reported on the treatment and outcomes of 146 AL patients who relapsed post ASCT [5]. However, specific regimen details and survival based on the regimens were not described. We present detailed treatments and outcomes of 292 patients who received a second line of therapy for their AL post ASCT and were not eligible for a second ASCT.

## Methods

This is a retrospective review of patients who were receiving a second line of therapy for their AL amyloidosis post ASCT between September 1997 and July 2019 at the Mayo Clinic, Rochester, Minnesota. Patients with multiple myeloma were excluded. Out of 719 patients who were treated with ASCT for AL amyloidosis, 343 (48%) were considered for a second line of therapy post ASCT. We excluded 51 patients, as 11 patients received treatment directed against a clonal lymphoid B-cell disorder (IgM amyloidosis), 16 patients proceeded directly to a second ASCT, 2 patients were involved in a clinical trial investigating the use of a monoclonal antibody for treating AL, and 22 patients did not receive any therapy. The remaining 292 patients received therapy against their clonal plasma cell disorder and were included in the analysis.

The diagnosis of AL was according to consensus criteria [6] and the revised Mayo 2012 staging system was applied at diagnosis [7], if possible. Starting a second line of therapy was decided by the treating physician even if the patient did not fulfill the exact criteria for progression (e.g., rising light chains but not reaching the defined level for progression) [8]. Details regarding the treatment received, response, and outcomes were documented. We only included patients who did not undergo a second ASCT and who received therapy against their clonal plasma cell disorder. The study was approved by the Mayo Clinic Institutional Review Board.

The patients were divided into six groups based on the treatment received. Patients who were treated with a combination of an alkylator, a PI, and dexamethasone (dex) or who received a PI with dex were in one group as (alkylator + PI ± dex, or PI ± dex). Those who received an alkylator, 2nd-generation (gen) IMiD, and dex or treated with a 2nd-gen IMiD, and dex were in a second group (alkylator + 2nd-gen IMiD ± dex or 2nd-gen IMiD ± dex). Patients treated with an alkylator with steroids or steroid monotherapy were in a third group (alkylator ± steroid or steroid monotherapy) and who received a combination of a 2nd-gen IMiD, PI, and dex were in the fourth group (2nd-gen IMiD + PI ± dex). Finally, those who were treated with an alkylator, thalidomide, and dex were in a fifth group (alkylator + thalidomide ± dex) and patients who had daratumumab combined with other drugs were in the sixth and final group (daratumumab-based).

The best hematological and organ responses to treatment after starting the second line were documented according to published criteria [8]. Event-free survival (EFS) was defined as the time from starting therapy post ASCT to disease progression or starting a new therapy. Progression-free survival (PFS) was defined as the time from starting a second line of treatment post ASCT to disease progression, changing therapy, or death, and OS was defined as the time from starting a second line of treatment post ASCT to death of any cause. Univariate analysis for OS was done for some important variables including age, bone marrow plasma cell % (BMPC %) at diagnosis, Mayo 2012 stage at diagnosis, number of organs involved, receiving induction therapy, and receiving conditioning with high-dose melphalan at diagnosis. Significant variables in the univariate analysis (*P* ≤ 0.1) were included in the multivariate analysis.

Statistical analysis was done using the JMP software (SAS Institute, Cary, NC) and the survival analysis was done using the Kaplan–Meier method, with *P* < 0.05 considered statistically significant.

## Results

We identified 292 patients who were receiving a second line of therapy for their AL amyloidosis post ASCT. The baseline characteristics of these patients are found in Table 1. The median age at starting the second line of therapy was 59 years, interquartile range (IQR) (53–64) and 64% were males. The details for the regimens received broken into six groups are provided in Table 2. Most patients (40%) were treated with an alkylator + PI ± dex or PI ± dex followed by an alkylator + 2nd-gen IMiD ± dex or 2nd-gen IMiD ± dex (26%) and an alkylator ± steroid or steroid monotherapy (19%). The remaining patients received a 2nd-gen IMiD + PI ± dex (6%), an alkylator + thalidomide ± dex (5%), and 4% had daratumumab-based therapy. Cyclophosphamide, bortezomib, dex (CyBorD), and bortezomib with dex (VD) were the most commonly used regimens in the alkylator + PI ± dex or PI ± dex group. Lenalidomide and dex (Rd) was the most commonly used regimen in the 2nd-gen IMiD ± dex group and melphalan with steroids was the most commonly used in the alkylator ± steroid or steroid monotherapy group. In the 2nd-gen IMiD + PI ± dex group, lenalidomide, bortezomib, and dexamethasone (VRd) was mostly used and in the daratumumab-based group, daratumumab ± dex was predominantly used.

**Table 1:** Baseline characteristics.

Variable	Cohort (*n* = 292)
Age at starting therapy (years) median, (IQR)	59 (53–64)
Male, *n* (%)	186 (64)
≥3 organ involved at diagnosis, *n* (%)	56 (19)
BMPCs ≥10% at diagnosis, *n* (%)	145 (50)
*t* (11;14)^a^ at diagnosis, *n* (%)	78 (37)
dFLC (mg/dl) at diagnosis median, (IQR)	17 (6–47)
Mayo 2012 stage at diagnosis^b^, *n* (%)	
1	93 (42)
2	69 (31)
3	37 (16)
4	24 (11)
NT-proBNP at starting therapy (pg/ml) median, (IQR)	455 (147–1640)
24 h urine protein at starting therapy (mg) median, (IQR)	1315 (135–5325)
Alkaline phosphatase at starting therapy (U/L) median, (IQR)	85 (64–111)

*IQR* interquartile range, *BMPC* bone marrow plasma cell, *dFLC* difference between involved and uninvolved free light chains.

^a^FISH was available in 213 patients.

^b^Staging was available in 223 patients.

**Table 2:** Details of treatment post ASCT.

Variable	Cohort (*n* = 292)
*Treatment post ASCT, n (%)*	
Alkylator + PI ± dex, or PI ± dex	118 (40)
CyBorD	64 (54)
Vd	51 (43)
Id	2 (2)
VMP	1 (1)
Alkylator + 2nd-gen IMiD ± dex, or 2nd-gen IMiD ± dex	77 (26)
Rd	59 (77)
Pd	10 (13)
CRd	8 (10)
Alkylator + steroid, or steroids monotherapy	55 (19)
Melphalan + steroids	39 (71)
Cyclophosphamide + steroids	2 (4)
dex	14 (25)
2nd-gen IMiD + PI ± dex	17 (6)
VRd	10 (59)
IRd	5 (29)
KPd	1 (6)
KRd	1 (6)
Alkylator + thalidomide ± dex	14 (5)
Td	13 (93)
CTd	1 (7)
Daratumumab-based	11 (4)
Dara ± dex	6 (55)
DPd	3 (27)
DRd	1 (9)
DVd	1 (9)

*ASCT* autologous stem cell transplantation, *PI* proteasome inhibitor, *dex* dexamethasone, *CyBorD* cyclophosphamide + bortezomib + dexamethasone, *Vd* bortezomib + dexamethasone, *Id* ixazomib + dexamethasone, *VMP* bortezomib + melphalan + dexamethasone, *gen* generation, *IMiD* immunomodulatory drug, *Rd* lenalidomide + dexamethasone, *Pd* pomalidomide + dexamethasone, *CRd* cyclophosphamide + lenalidomide + dexamethasone, *VRd* bortezomib + lenalidomide + dexamethasone, *IRd* ixazomib + lenalidomide + dexamethasone, *KPd* carfilzomib + pomalidomide + dexamethasone, *KRd* carfilzomib + lenalidomide + dexamethasone, *Td* thalidomide + dexamethasone, *CTd* cyclophosphamide + thalidomide + dexamethasone, *Dara* daratumumab, *DPd* daratumumab + pomalidomide + dexamethasone, *DRd* daratumumab + lenalidomide + dexamethasone, *DVd* daratumumab + bortezomib + dexamethasone.

The median duration of treatment was 6 months, IQR (3.5–12). The alkylator + 2nd-gen IMiD ± dex or 2nd-gen IMiD ± dex group had the longest median treatment duration of 10 months, IQR (5–20), followed by the 2nd-gen IMiD + PI ± dex group and the alkylator + PI ± dex or PI ± dex group (6 months, IQR: 4–13) and (6 months, IQR: 3–10), respectively. The median duration of treatment was 5.5 months, IQR (3–10) and 6 months, IQR (3–8) in the alkylator + thalidomide ± dex and the alkylator ± steroid or steroid monotherapy groups, respectively. The daratumumab-based group had a median treatment duration of 5.5 months, IQR (3–7).

**Fig. 1:**
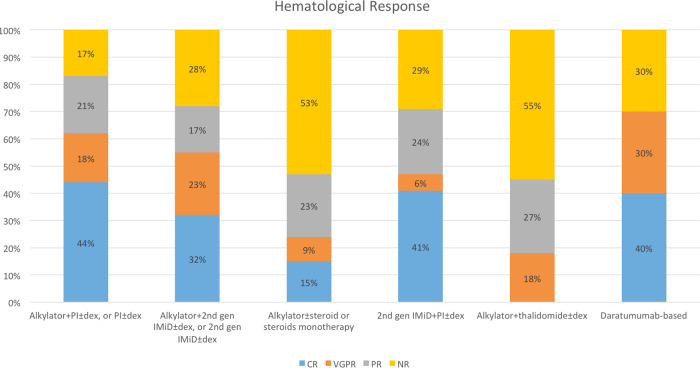
Hematological response in evaluable patients by the regimen received. PI proteasome inhibitor, dex dexamethasone, gen generation, IMiD immunomodulatory drug, CR complete response, VGPR very good partial response, PR partial response, NR no response.

Some patients had missing lab information and could not be evaluated for response (*n* = 31), of which 14 were in the alkylator + PI ± dex, or PI ± dex group, 8 were in the alkylator with steroids or steroid monotherapy, 5 were in the alkylator + 2nd-gen IMiD ± dex or 2nd-gen IMiD ± dex group, 3 were in the alkylator + thalidomide ± dex, and 1 was in the daratumumab-based group. The overall hematological response rate (ORR) in evaluable patients was 70%, with 33% achieving complete response (CR), 17% achieving a very good partial response (VGPR), and 20% achieving a partial response (PR). The hematological response in evaluable patients based on the different treatment groups is displayed in Fig. 1. The ORR was 83% in the alkylator + PI ± dex or PI ± dex group, 72% in the alkylator + 2nd-gen IMiD ± dex or 2nd-gen IMiD ± dex group, 71% in the 2nd-gen IMiD + PI ± dex group, 70% in the daratumumab-based group, 45% in the alkylator + thalidomide ± dex group, and 47% in the alkylator ± steroid or steroid monotherapy group. The rate of achieving CR or VGPR was 70% among the daratumumab-based group, 62% in the alkylator + PI ± dex or PI ± dex group, 55% in the alkylator + 2nd-gen IMiD ± dex or 2nd-gen IMiD ± dex group, 47% in the 2nd-gen IMiD + PI ± dex group, 24% in the alkylator ± steroid or steroid monotherapy group, and 18% in the alkylator + thalidomide ± dex group (*P* < 0.0001).

Assessment for the cardiac response was available in 93 patients, of whom 24 (26%) had a response, and the remaining patients either did not respond or progressed. Of these evaluable patients, 34 were treated with an alkylator + PI ± dex, or PI ± dex and 12 (35%) responded, 29 received an alkylator + 2nd-gen IMiD ± dex or 2nd-gen IMiD ± dex, with 6 (21%) responding, 20 had a treatment with an alkylator ± steroid or steroid monotherapy, with 2 (10%) responding, 5 were treated with a 2nd-gen IMiD + PI ± dex and 3 (60%) had a response, 2 were treated with a daratumumab-based regimen with 1 patient (50%) responding, and 3 had an alkylator + thalidomide ± dex with none responding.

For renal response assessment, 91 patients were evaluable with 53 (58%) showing response. Forty patients were treated with an alkylator + PI ± dex, or PI ± dex, with 60% response rate and 25 patients received either an alkylator + 2nd-gen IMiD ± dex or a 2nd-gen IMiD ± dex with 56% responding in both regimens. Nineteen patients had a treatment with an alkylator ± steroid or steroid monotherapy with a response rate of 58%. Three patients were treated with a 2nd-gen IMiD + PI ± dex, and also three were treated with an alkylator + thalidomide ± dex with a response rate of 67% in both groups. Finally, one patient received a daratumumab-based regimen and did not respond.

Only 36 patients were evaluable for liver response and 9 (25%) responded. Thirteen patients were treated with an alkylator ± steroid or steroid monotherapy and two (15%) responded. Only one (17%) of six patients treated with an alkylator + PI ± dex, or PI ± dex had a response, as well as three (33%) of nine patients treated with an alkylator + 2nd-gen IMiD ± dex or 2nd-gen IMiD ± dex. Three patients were treated with either a 2nd-gen IMiD + PI ± dex or an alkylator + thalidomide ± dex and in either groups, only one patient (33%) responded. Finally, two patients were treated with a daratumumab-based therapy and one patient (50%) responded.

When evaluating the response based on the Mayo 2012 stage at diagnosis, the rate of CR/VGPR in patients with Mayo 2012 stage I/II was 67% for the daratumumab-based group, 62% in the alkylator + PI ± dex, or PI ± dex group, 56% in the alkylator + 2nd-gen IMiD ± dex group, 44% in the 2nd-gen IMiD + PI ± dex group, 25% in the alkylator + thalidomide ± dex group, and 7% in the alkylator ± steroid or steroid monotherapy. In patients with Mayo 2012 stage III/IV, the rate of CR/VGPR was 75% in the alkylator + PI ± dex, or PI ± dex group, 67% in the daratumumab group, 56% in the alkylator + 2nd-gen IMiD ± dex group, 50% in the 2nd-gen IMiD + PI ± dex group, 43% in the alkylator ± steroid or steroid monotherapy, and 33% in the alkylator + thalidomide ± dex group. The OS based on the regimen in patients with Mayo 2012 stage I/II is shown in Supplementary Fig. 1A, and in patients with Mayo 2012 stage III/IV in Supplementary Fig. 1B.

We also evaluated the induction therapy (if any) given before ASCT in our cohort. Most of our patients did not receive any therapy or were treated with steroids only before ASCT (71%). The remaining received a PI-based regimen (11%), IMiD-based (6%), alkylator-based (6%), IMiD + PI (4%), or other therapy (2%). Of patients who were treated with an alkylator + PI ± dex, or PI ± dex after ASCT, 8% received induction therapy with a PI-based regimen before ASCT and 4% were treated with an induction therapy that included an IMiD + PI. Patients who were treated with a 2nd-gen IMiD + PI ± dex after transplant received mostly an induction with a PI-based regimen (29%) or an IMiD-based regimen (18%), but none received an IMiD + PI regimen before ASCT. A PI-based regimen was the most commonly used induction regimen before ASCT (18%) for patients who received an alkylator + 2nd-gen IMiD ± dex or 2nd-gen IMiD ± dex after ASCT. For patients who received daratumumab-based therapy, the induction regimen before ASCT was mainly a PI based (36%) or a combination of an IMiD and a PI (27%).

**Fig. 2:**
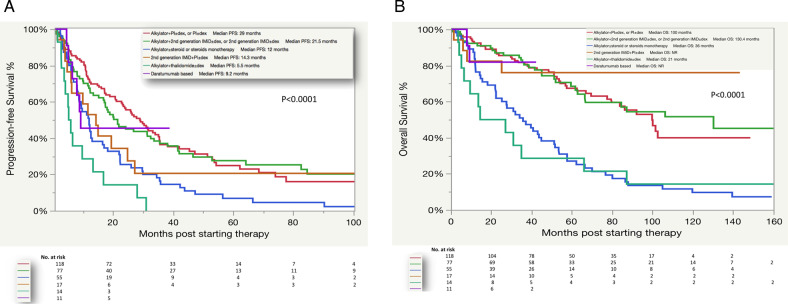
Survival of the whole cohort by the different regimens. PFS (**A**) and OS (**B**). PFS progression-free survival, OS overall survival, PI proteasome inhibitor, dex dexamethasone, IMiD immunomodulatory drug, NR not reached.

**Fig. 3:**
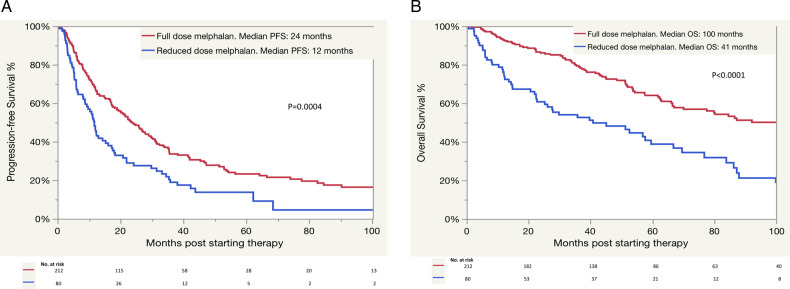
Survival according to the melphalan dose received at diagnosis. PFS (**A**) and OS (**B**). PFS progression-free survival, OS overall survival.

The median follow-up was 81 months (95% confidence interval (CI): 68–94). The median PFS and OS for the whole cohort were 19.4 months (95% CI: 16–24) and 78 months (95% CI: 62–92), respectively. The PFS and OS for the different treatment groups are shown in Fig. 2A, B, respectively. The median PFS was 29 months (95% CI: 23–35) for the alkylator + PI ± dex or PI ± dex group, 21.5 months (95% CI: 17–33.6) for the alkylator + 2nd-gen IMiD ± dex or 2nd-gen IMiD ± dex group, 14.3 months (95% CI: 5–25 months) for the 2nd-gen IMiD + PI ± dex group, 12 months (95% CI: 8.3–16.5) for the alkylator ± steroid or steroid monotherapy group, 9.2 months (95% CI: 5–not reached (NR)) for the daratumumab-based group, and 5.5 months (95% CI: 3–13.3 months) for the alkylator + thalidomide ± dex group (*P* < 0.0001). When calculating the EFS based on the different regimens, the median EFS was similar to PFS across all regimens except, the alkylator + thalidomide ± dex group (9.7 vs. 5.5 months) and the daratumumab-based group (NR vs. 9.2 months). The median OS was NR (95% CI: 25.2–NR) for the 2nd-gen IMiD + PI ± dex group, NR (95% CI: 9.2–NR) for the daratumumab group, 130.4 months (95% CI: 66.5–NR) in the alkylator + 2nd-gen IMiD ± dex or 2nd-gen IMiD ± dex group, 100 months (95% CI: 80–NR) for the alkylator + PI ± dex or PI ± dex group, 36 months (22.4–51.3) for the alkylator ± steroid or steroid monotherapy group, and 21 months (5–66) for the alkylator + thalidomide ± dex group (*P* < 0.0001). We also examined the effect of the conditioning dose of melphalan that was used before ASCT (200 vs.140 mg/m^2^) on the PFS and OS following starting a second line of therapy (Fig. 3A, B). Interestingly, patients who received full-dose melphalan had better PFS and OS than patients who received reduced dosing (median PFS: 24 vs. 12 months, *P* = 0.0004 and median OS: 100 vs. 41 months, *P* < 0.0001), respectively. Univariate and multivariate analysis for OS was done (Table 3). In the multivariate analysis, receiving high-dose melphalan for conditioning was an independent predictor of survival.

**Table 3:** Univariate and multivariate analysis for OS.

Variable	Univariate analysis		Multivariate analysis	
	HR (95% CI)	*P*	HR (95% CI)	*P*
Age >65 at relapse	1.4 (0.99–1.97)	**0.05**	1.2 (0.78–1.9)	0.3
Mayo 2012 stage (III/IV) at diagnosis	2.4 (1.58–3.6)	**<0.0001**	1.6 (1.01–2.6)	**0.04**
BMPC ≥10% at diagnosis	1.3 (0.93–1.81)	**0.1**	1.14 (0.75–1.75)	0.5
Organs involved >2 at diagnosis	1.05 (0.70–1.56)	0.8	–	–
Conditioning melphalan 200 mg/m^2^ at diagnosis	0.43 (0.3–0.6)	**<0.0001**	0.45 (0.27–0.74)	**0.001**
Receiving induction therapy	1.23 (0.88–1.7)	0.2	–	**–**

*OS* overall survival, *HR* hazard ratio, *CI* confidence interval, *BMPC* bone marrow plasma cell.

The bold values indicate a statistically significant result.

## Discussion

The management of patients who relapse post ASCT for AL can be challenging, as the literature is limited and the treatment decisions are based on multiple factors, including but not limited to, patient fitness, specific organ involvement, previous therapy received, and toxicity. We present the largest cohort of patients who required additional therapy post ASCT for their AL. In our study, 292 patients received six classes of therapies with a median duration of treatment of 6 months. The ORR was ≥70% in patients treated with novel therapy with >45% achieving CR/VGPR. The median OS was >8 years for patients treated with novel agents.

Multiple agents can be used for the treatment of relapsed AL amyloidosis. Bortezomib has been evaluated in retrospective [9] as well as small prospective trials [10, 11]. In phase I/II trial [10], in which bortezomib was given once weekly or twice weekly in patients with relapsed/refractory AL amyloidosis, the ORR was around 70%, with CR rates ranging between 24 and 38%. The median OS was 62 months in patients receiving once-weekly dosing. A retrospective study evaluated response rates and outcomes of patients receiving bortezomib [9] and 76 patients received it for relapsed/refractory disease with an ORR of 68%, and CR rates of 20%. In another retrospective review of 43 patients who received CyBorD for AL amyloidosis [12], 23 patients had relapsed disease and 74% had responded with 22% achieving CR. In a phase 3 trial for patients with relapsed/refractory AL amyloidosis who were randomized to ixazomib + dex or physician’s choice, the hematological response rate in the ixazomib + dex group was 53% with 26% achieving CR and a median duration of hematological response of 46.5 months [13]. In our cohort, we have treated 117 patients with an alkylator + PI ± dex, or PI ± dex, with an ORR of 83% with 62% achieving CR/VGPR and a median OS of 8 years.

Patients with AL amyloidosis usually have multiple organ involvement that limits the ability to use a combination of an IMiD and a PI, both of which can cause cardiac toxicity. In our cohort, only 6% of patients were treated with this combination with an ORR of 71% with 47% having CR/VGPR and a median OS that was NR. In a retrospective study that evaluated 40 patients with relapsed AL who received ixazomib, lenalidomide, and dex, the ORR was 64% with 46% achieving CR/VGPR [14]. The median PFS was17 months and the median OS was 29 months.

Our findings in addition to the available evidence suggest that having a PI-based therapy is critical in the management of patients with AL who require subsequent therapies. Bortezomib and ixazomib are generally well tolerated and can be combined with other therapy for patients who require subsequent therapy post ASCT. The combination of a PI with an alkylator or an IMiD results in deep responses and excellent survival outcomes. Thus, this class should be highly considered when choosing a regimen for patients who require therapy post ASCT.

In a phase II trial of patients with AL amyloidosis, lenalidomide ± dex was tested and 13 patients (59%) were previously treated for their AL amyloidosis, with an ORR of 38% [15]. In another phase II trial [16], 31 patients (91%) were previously treated for their AL amyloidosis and lenalidomide ± dex showed an ORR of 67%, with CR rates of 29% in evaluable patients. Palladini et al. [17] described 24 patients with relapsed/refractory AL who received lenalidomide ± dex with an ORR of 41% and a median survival of 14 months. Cyclophosphamide in addition to lenalidomide and dex has been evaluated in patients with AL amyloidosis [18, 19], and in patients with relapsed AL amyloidosis, the ORR was 58% [18]. Pomalidomide has also been evaluated in 33 patients with relapsed AL amyloidosis with a 48% ORR, a median PFS of 14 months, and a median OS of 27.9 months [20]. In our patient population treated with an alkylator + 2nd-gen IMiD ± dex or 2nd-gen IMiD ± dex, the ORR was 73% with 56% having CR/VGPR and a median OS of 10.8 years.

The monoclonal antibody against CD38, daratumumab, has significant activity in patients with relapsed or refractory AL amyloidosis [21–24]. In the largest phase II trial of 40 patients [21], daratumumab was given as a single agent with an ORR of 55%, with 47.5% achieving VGPR or better. The median PFS was 24.8 months and the median OS was NR. In another phase II trial [22], 22 patients received daratumumab with an ORR of 90%, mostly being VGPR or better (86%) with a median PFS of 28 months. In retrospective reviews, ORR >75% have been documented [23, 24], and the use of daratumumab in combination with other therapy (pomalidomide + dex, lenalidomide + dex, and bortezomib + dex) have been described [23]. Although no direct comparison could be made between those who received daratumumab combined with other therapy and those who received it as a single agent, the ORR was noted to be higher for those who received the combination therapy 88% vs. 78% [23]. Based on the available literature and our findings, we recommend that patients with AL amyloidosis that require additional therapy post ASCT receive novel agents-based therapy.

The OS of our patients was above what is expected for patients who had a relapse of their AL and were not eligible for ASCT upfront. The median OS was 7 years for the whole cohort, with a median survival that is NR in some patients treated with novel therapy. The fact that these patients were eligible for ASCT upfront makes them more likely to be fit for future therapy with very few stage IV patients. The introduction of novel agents has improved the survival of AL. The excellent survival for patients who relapse post ASCT for AL amyloidosis has also been reported with a median OS of 8.5 years [3]. Being able to receive full-dose melphalan in our population before ASCT was associated with better survival, even after requiring additional therapy post ASCT. The median OS (from starting a second line) for patients who received full-dose melphalan was 8.5 years compared to 3.8 years for patients who received the reduced dose. This is an important finding emphasizing the critical role of high-dose melphalan in improving survival even after a relapse. This has also been previously reported in a previous study with a median OS of 15 years vs. 4.2 years for patients who received full and reduced doses of melphalan, respectively [3].

Our study is limited by the nature of retrospective studies. Our patients received therapy over a long period and the regimens were variable. However, we present the largest cohort of patients treated with a second line for their AL after ASCT. The survival of these patients has improved especially with the use of novel therapy.

## Supplementary information

Legend for supplementary figure

Supplementary Figure 1. Overall survival based on the regimen and Mayo 2012 stage. Mayo 2012 stage I/II (A) and Mayo 2012 stage III/IV (B).
