# Supplementary material for: Treatment and outcomes of patients with light chain amyloidosis who received a second line of therapy post autologous stem cell transplantation
Source: Blood Cancer J. 2022 Apr 11;12(4):59. doi: 10.1038/s41408-022-00655-z (PMC9001695; doi:10.1038/s41408-022-00655-z)
Supplement: Supplementary file 1 — Legend for supplementary figure [file 41408_2022_655_MOESM1_ESM.docx]

Supplementary Figure 1. OS based on the regimen in patients A. with Mayo 2012 stage I/II and in B. patients with Mayo 2012 stage III/IV .
