# Supplementary figures and images for: Treatment and outcomes of patients with light chain amyloidosis who received a second line of therapy post autologous stem cell transplantation
Source: Blood Cancer J. 2022 Apr 11;12(4):59. doi: 10.1038/s41408-022-00655-z (PMC9001695; doi:10.1038/s41408-022-00655-z)

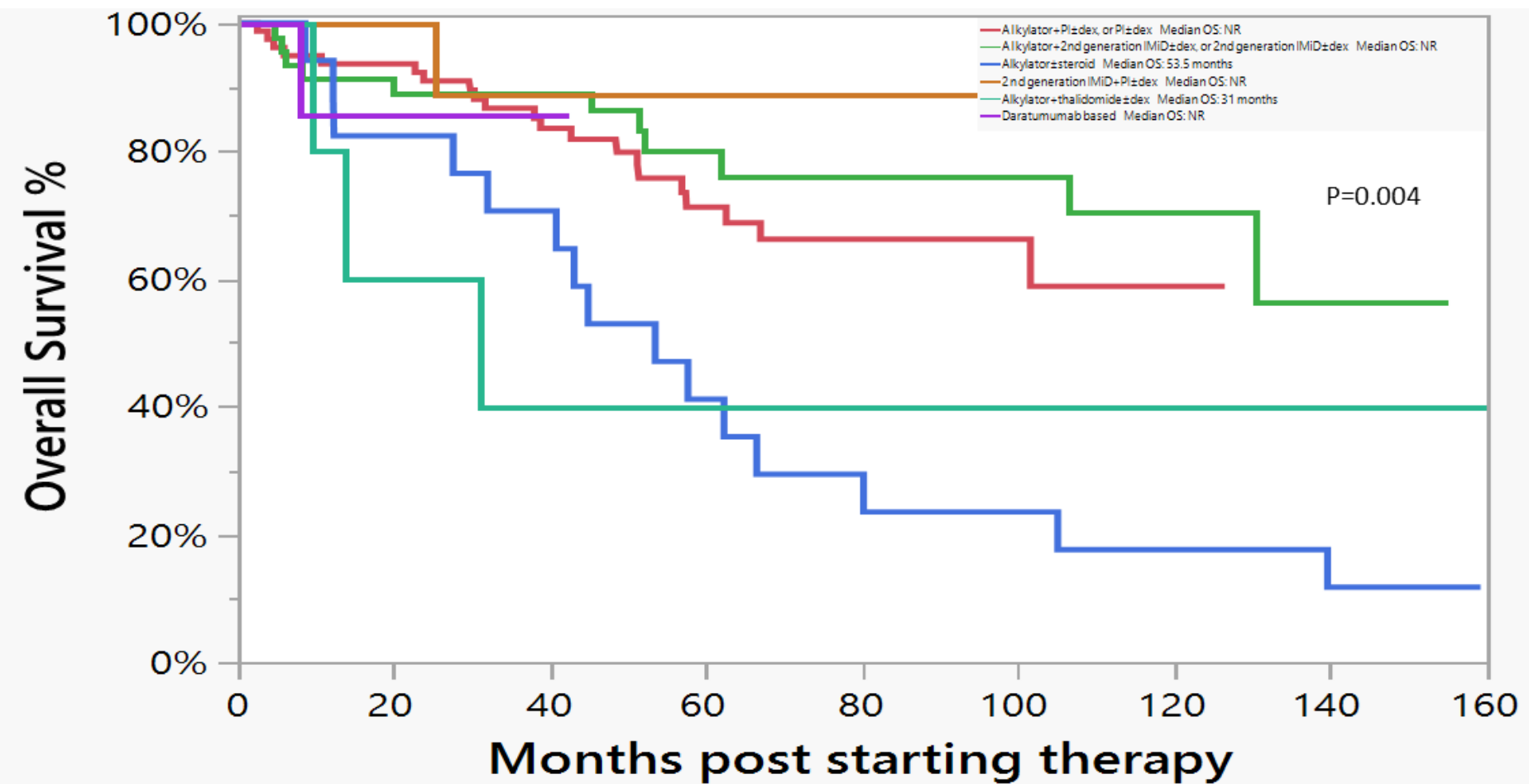

| No. at risk |    |    |    |    |    |   |   |   |   |
|-------------|----|----|----|----|----|---|---|---|---|
| 78          | 71 | 52 | 30 | 20 | 9  | 1 |   |   |   |
| 46          | 40 | 37 | 20 | 16 | 14 | 9 | 3 |   |   |
| 17          | 14 | 12 | 7  | 5  | 4  | 3 | 2 | 1 |   |
| 9           | 9  | 6  | 2  | 2  | 2  | 2 | 2 | 2 | 2 |
| 5           | 3  | 2  | 2  | 2  | 2  | 2 | 2 | 2 | 2 |
| 7           | 4  | 1  |    |    |    |   |   |   |   |

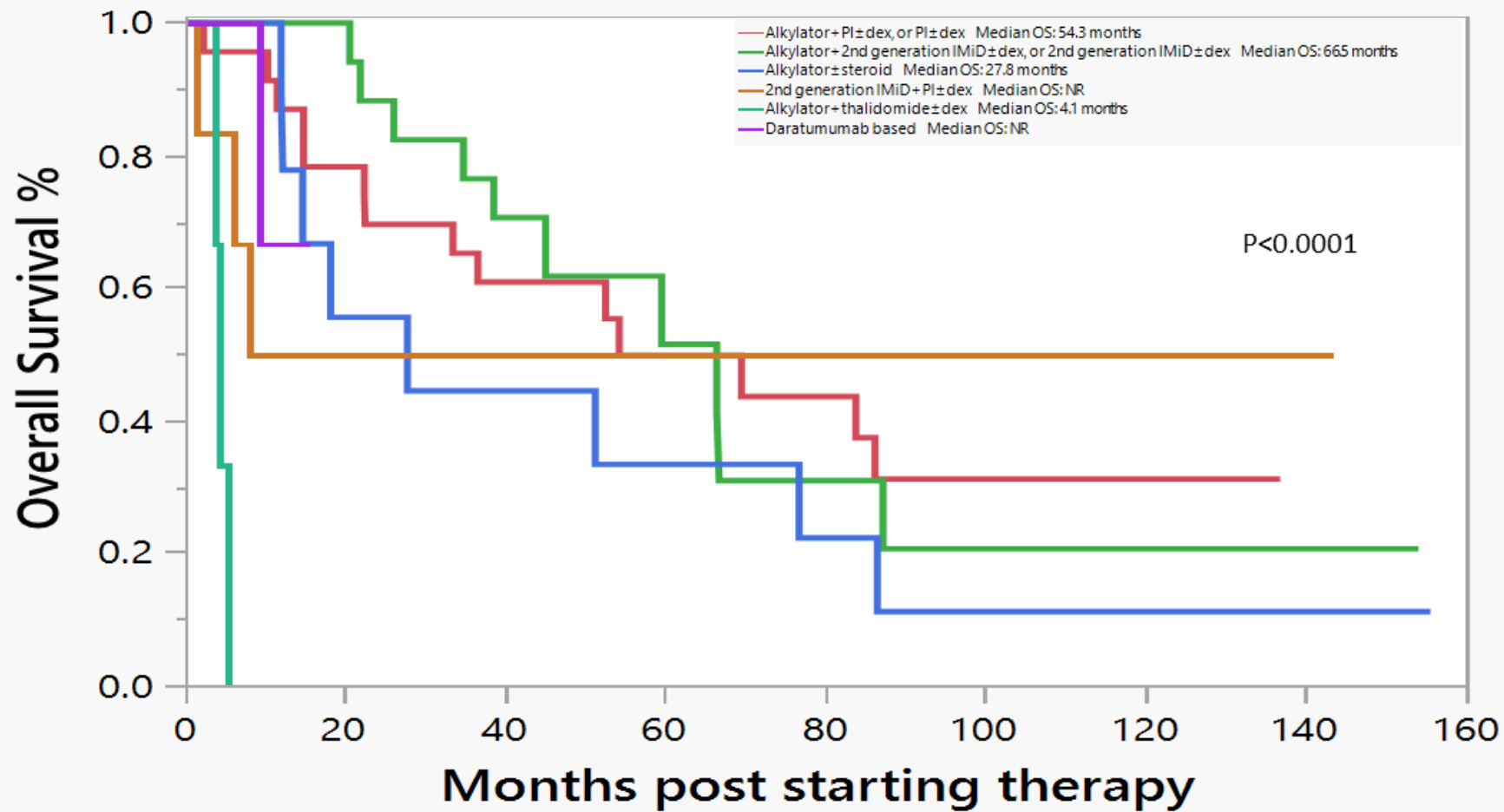

No. at risk

|    |    |    |   |   |   |   |   |
|----|----|----|---|---|---|---|---|
| 23 | 18 | 14 | 9 | 7 | 2 | 1 |   |
| 17 | 17 | 12 | 6 | 3 | 2 | 1 |   |
| 9  | 5  | 4  | 3 | 2 | 1 | 1 | 1 |
| 6  | 3  | 2  | 2 | 1 | 1 | 1 | 1 |
| 3  |    |    |   |   |   |   |   |
| 3  |    |    |   |   |   |   |   |

Supplement: Supplementary file 2 — Supplementary Figure 1. Overall survival based on the regimen and Mayo 2012 stage. Mayo 2012 stage I/II (A) and Mayo 2012 stage III/IV (B). [file 41408_2022_655_MOESM2_ESM.pdf]
